# Congenital anomalies identified during early childhood in NICU infants with bilateral hearing loss: a multi-centre longitudinal cohort study

**DOI:** 10.3389/fnins.2026.1836937

**Published:** 2026-07-22

**Authors:** Sally K. Thornton, Hayma Moosan, Dulip Jayasinghe, Karen R. Willis, Katherine Martin, Alexandra Jayasinghe, Mohamad Amin Pourhoseingholi, Derek J. Hoare

**Affiliations:** 1 Hearing Sciences, Mental Health and Clinical Neurosciences, School of Medicine, The University of Nottingham, Nottingham, United Kingdom; 2 NIHR Nottingham Biomedical Research Centre, Nottingham, United Kingdom; 3 Neonatal Intensive Care Unit, City Hospital Campus, Nottingham University Hospitals, Nottingham, United Kingdom; 4 Audiology, Nottingham University Hospitals, Nottingham, United Kingdom; 5 Child Development Centre, City Hospital Campus, Nottingham University Hospitals, Nottingham, United Kingdom; 6 The Medical School, Newcastle University, Newcastle-Upon-Tyne, United Kingdom

**Keywords:** bilateral hearing loss, congenital anomalies, markers, neonatal intensive care, neurodevelopmental impairment

## Abstract

**Introduction:**

Infants requiring neonatal intensive care are exposed to a range of biological insults during critical periods of brain development, increasing the risk of both bilateral hearing loss (BHL) and neurodevelopmental impairment (NDI). While hearing loss is often seen as a sensory deficit, emerging evidence suggests it may reflect broader disturbances in neurodevelopment. The extent to which multisystem abnormalities associated with BHL are documented at birth or identified during early childhood follow-up remains unclear. This study investigated the temporal relationship between congenital anomalies, hearing loss, and neurodevelopmental outcomes in NICU populations.

**Methods:**

A retrospective multi-centre cohort study was conducted across two UK tertiary NICUs. Infants with confirmed permanent BHL were matched to NICU controls without hearing loss based on gestational age, birthweight and sex. Congenital anomalies were assessed at birth and at 2-years using standardised clinical classifications. Neurodevelopmental outcomes at 2-years were categorised as normal-mild or moderate-severe delay. Stratified analyses by site were performed, with pooled associations estimated using Mantel-Haenszel methods. Multivariable logistic regression was used to examine independent predictors of NDI.

**Results:**

At birth, results indicate there was no significant difference in congenital anomaly prevalence between infants with BHL and matched controls (Mantel-Haenszel χ^2^ = 1.17, *p* = 0.28; OR 1.47, 95% CI 0.80–2.71). By 2-years, however, children with BHL demonstrated a significantly greater burden of congenital anomalies across both centres (pooled χ^2^ = 20.47, *p* < 0.001), with over four-fold increased odds compared with controls (OR 4.43, 95% CI 2.39–8.20). Differences were most pronounced in abnormalities of the nervous system. In multivariable models, BHL was associated with substantially increased odds of moderate-to-severe NDI (OR 13.89, *p* < 0.001), with additional contributions from congenital anomalies (OR 2.57, *p* = 0.023).

**Discussion:**

These findings suggest that BHL in NICU populations may be associated with broader developmental vulnerability rather than isolated auditory pathology. Longitudinal follow-up may therefore be important for identifying congenital anomalies that are not clinically apparent or documented in the neonatal period and informing more targeted developmental surveillance.

## 1 Introduction

Survival among infants requiring neonatal intensive care has improved substantially over the past several decades. However, neonatal intensive care unit (NICU) admission remains associated with an increased risk of long-term neurodevelopmental impairment (NDI), particularly among infants born very preterm or with severe neonatal illness (Chung et al., 2020). In contemporary cohorts of extremely preterm infants assessed in early childhood, moderate-to-severe NDI has been reported in approximately 10%–26% of survivors, with rates exceeding 40% among those born before 25 weeks’ gestation in some studies (Draper et al., 2020). Even among children without severe disability, meta-analyses demonstrate persistent differences in cognitive ability, executive functioning, and processing speed compared with term-born peers (van Houdt et al., 2019). These findings underscore the need for early, clinically accessible markers of neurodevelopmental vulnerability in NICU populations. Current clinical markers are insufficient as NDI is often not identified until early childhood.

Hearing loss represents one of the most clinically significant sensory complications in NICU survivors. Permanent childhood hearing loss, even when mild, can disrupt early speech and language acquisition and has lasting consequences for educational attainment, social participation, and employment opportunities (Ching et al., 2018; Lieu, 2018). The introduction of universal newborn hearing screening (UNHS) has enabled earlier detection in many countries. Population estimates suggest that permanent bilateral hearing loss (BHL) occurs in approximately 1.3 per 1,000 live birth; however, prevalence is substantially higher in NICU populations, with estimates up to tenfold greater than in the general population, reflecting different inclusion criteria, higher-risk clinical profiles and increased exposure to neonatal risk factors (Fortnum and Davis, 1997; Wroblewska-Seniuk et al., 2017). Expanded genomic sequencing combined with hearing screening may be effective at detecting hearing loss among NICU cases (Zhu et al., 2022). Despite advances in early detection and intervention, children with hearing loss remain at increased risk of broader developmental difficulties (de Jong et al., 2024).

The mechanisms linking NICU admission and hearing loss are complex and multifactorial. Established risk factors include congenital infection, genetic conditions, and structural anomalies affecting the auditory system, as well as postnatal exposures such as hypoxic–ischaemic injury, severe infection, prolonged respiratory support, exposure to ototoxic medications (Arribas et al., 2024; Luo et al., 2026) and metabolic instability during critical periods of brain maturation (Korver et al., 2017; Thangavelu et al., 2019; Chant et al., 2023; Verstappen et al., 2023; Han et al., 2024; Thornton et al., 2024; Shearer et al., 2025). Importantly, many of these exposures are also recognised as risk factors for broader NDI in NICU survivors. Conditions such as intraventricular haemorrhage, periventricular leukomalacia, bronchopulmonary dysplasia, and systemic infection are consistently associated with adverse cognitive and behavioural outcomes in early childhood (Marlow et al., 2005; Volpe, 2009; Groenendaal and Uiterwaal, 2012; Moore et al., 2012).

These overlapping risk profiles raise the possibility that hearing loss in NICU populations may not always represent an isolated sensory disorder but instead may occur as part of a broader pattern of developmental vulnerability.

Congenital anomalies may represent a key mechanistic link between hearing loss and broader neurodevelopmental outcomes in NICU populations. Structural anomalies affecting multiple organ systems can reflect disruptions in early embryological development and may influence both auditory and neurodevelopmental pathways. Previous work has reported increased rates of congenital anomalies and developmental impairment in NICU subgroups with hearing loss, including those with unilateral hearing loss (Horrocks et al., 2022; Moosan et al., 2024). Major congenital anomalies are present in approximately 2%–3% of live births in the general population (Dolk et al., 2010; Morris et al., 2019) but are more common among children with permanent childhood hearing impairment, particularly in syndromic or genetically mediated conditions (Korver et al., 2017). Some congenital disorders, such as Down syndrome or CHARGE syndrome, are characterised by co-occurring hearing impairment and neurodevelopmental abnormalities affecting multiple organ systems (Laws and Hall, 2014; van Ravenswaaij-Arts and Martin, 2017). These findings suggest that the co-occurrence of hearing loss, congenital anomalies, and NDI may be consistent with shared developmental origins that link auditory and neurodevelopmental pathways rather than independent consequences of prematurity alone.

Despite these associations, the extent to which congenital anomalies contribute to neurodevelopmental outcomes in NICU children with hearing loss remains unclear. Existing studies have largely examined hearing loss or NDI in isolation, with limited investigation of their co-occurrence within NICU populations with confirmed hearing loss.

This study aimed to investigate the relationship between congenital anomalies and NDI in NICU graduates with confirmed BHL. We compared the prevalence and pattern of congenital anomalies between children with BHL and matched NICU controls and examined their association with NDI at 2-years of age.

## 2 Materials and methods

This study used data from the Nottingham NICU research database (NEAT) and was approved for secondary analysis of routinely collected data by the South-Central Berkshire Research Ethics Committee (REC project ID: 292263).

### 2.1 Study design and population

This was a longitudinal, multi-centre cohort study which documented congenital anomaly data at birth and at 2-years and developmental outcome data from two tertiary neonatal centres in Nottingham (Site 1) and one in Newcastle (Site 2). The primary objective was to examine congenital anomalies and developmental outcomes in NICU graduates with BHL.

No formal sample size calculation was performed, as this was a retrospective cohort study including all eligible children with bilateral hearing loss (BHL) identified during the study periods (2008–2020 at Site 1 and 2010–2022 at Site 2). The final sample size therefore reflects the available clinical population rather than investigator-selected recruitment targets.

#### 2.1.1 Inclusion criteria

Children were eligible for inclusion if they had both a history of NICU admission and confirmed permanent BHL diagnosed following the UNHS, confirmed by auditory brainstem response (ABR) testing and paediatric audiology assessment. BHL was defined according to British Society of Audiology (BSA) criteria as ≥21 dB HL in both ears (Current Guidance - The BSA, 2018). Data for children with unilateral hearing loss from this database have been reported previously (Horrocks et al., 2022).

A matched control group of NICU graduates without hearing loss was included. Cases and controls were matched within each site on gestational age, birthweight, and sex, as key clinical determinants of hearing and neurodevelopmental outcomes. Exact temporal matching (e.g., date of birth or admission) was not performed due to feasibility constraints and the risk of substantially reducing the number of eligible pairs. The control group were matched 1:1 on birthweight (+10 g), gestational age (+1 week), sex, and duration of their stay in the NICU (<48 vs. ≥48 h). If an exact match could not be identified, birthweight matching criteria were incrementally relaxed in 10 g steps up to ± 50 g, followed by gestational age adjustment in 1 week increments up to ± 2 weeks. In two cases (one per site), matching on gestational age and birthweight was not possible even following adjustment; the matched control therefore differed on sex.

#### 2.1.2 Exclusion criteria

Control cases were excluded if they had been referred following NHSP screening or had any evidence of acquired hearing loss at the time of data collection. The data from *n* = 5 children with BHL and a NICU admission were excluded as they did not survive until their 2-year follow-up.

### 2.2 Neonatal data collection

Neonatal birth data were extracted for both groups. Data entry onto NICU Badgernet (Clevermed, Edinburgh) database was completed by a member of the NICU medical team.

Data were extracted from electronic health records (including neonatal and hospital information systems) and supplemented with paper records where necessary. At both sites, Badgernet served as the primary route for retrieving an individual’s 2-year developmental follow-up and demographic data (sex, gestational age, and birthweight).

Principal diagnoses at birth and at 2-years were recorded, together with any documented genetic diagnosis.

#### 2.2.1 Audiological data

Permanent BHL in this study, encompasses all types of clinically confirmed BHL first identified through newborn screening and then confirmed through follow-up pathways. Hearing threshold data were taken from their most recent audiological report from a paediatric audiologist. Depending on age and stage of development, hearing data were taken from the available pure-tone audiogram or play and visual reinforcement audiometry. Hearing data were also extracted from air and bone conduction tests (ABR - Biologic Navigator Pro or Medelec Synergy). According to BSA guidelines, the degree of BHL in children is classified based on the average hearing threshold of the better ear only. This calculation uses the pure tone average (PTA) at frequencies 0.5, 1, 2, and 4 kHz, or 0.25–4 kHz in some protocols. Importantly, hearing loss classification in this study was based on longitudinal audiological assessment, incorporating diagnostic ABR alongside subsequent behavioural testing (PTA/VRA), reflecting routine clinical pathways rather than a single modality.

### 2.3 Congenital anomalies and 2-year developmental outcomes

Congenital anomalies were systematically recorded after birth during the neonates’ NICU stay and again at the 2-year follow up. To ensure consistency in quantification, multiple anomalies affecting the same organ or organ system were counted as a single entity for analysis. Congenital anomaly and NDI data were extracted by a clinical researcher, checked by a second research fellow and verified by a consultant neonatologist. All suspected anomalies were reviewed by a consultant neonatologist to support accurate identification and classification. Anomalies were categorised according to established coding frameworks (EUROCAT and ICD-10), with further sub-grouping by organ/system based on the pre-defined study categorisation (Bergman et al., 2024). The descriptions of anomalies in Supplementary Material (5) indicate which anomalies were included. A neonatal consultant aided in anomaly classification, and the classification was undertaken by consensus. Sensory losses (hearing, eyesight) were not counted unless there was a physical abnormality associated with the deficit.

Congenital anomalies were recorded based on clinical documentation at two timepoints (neonatal period and 2-year follow-up). The timing of diagnosis varied according to clinical presentation and investigation pathways, with some anomalies identified only during follow-up rather than in the neonatal period.

Aetiological investigations, including genetic testing (see Supplementary Table 4), were performed on a clinically indicated basis rather than as part of a standardised study protocol and were therefore not available for all participants. Variant classifications were extracted from clinical records and were not reinterpreted or reclassified for this study; variant-level detail was not consistently available due to the retrospective nature of data extraction.

Two-year developmental outcome data were extracted from notes that had been entered by Paediatric Consultants who are specialists in developmental follow-up. Children were diagnosed with developmental impairment if their developmental skills fell two standard deviations or more below the population mean in two or more developmental domains. Degree of developmental delay was scored using the developmental quotient (developmental age/chronological age × 100). Where available, standardised scores from the Bayley Scales of Infant and Toddler Development (Bayley-III) were also used for developmental assessments. 2-year follow-up data were extracted from clinical records and included developmental, behavioural, social, and medical outcomes. Children were classified as having no documented developmental impairment where follow-up records indicated typical development or where no developmental concerns, referrals, or support needs were documented. Developmental status at 2-years of age was recorded and categorised as normal development or mild, moderate, or severe impairment based on documentation in follow-up assessments by consultant paediatrician. For analysis, outcomes were grouped as normal–mild or moderate–severe NDI.

### 2.4 Missing data

Missing data were evaluated for all study variables. The overall proportion of missing data was low (<5%), and patterns of missingness did not suggest systematic bias. Analyses were performed using available case analysis, with participants only included where complete data were available for the variables of interest.

### 2.5 Statistical analysis

Statistical analyses were performed in SPSS (version 28.0.0.0, IBM Corp., NY, USA). Descriptive statistics were used to summarise cohort characteristics.

For matched comparisons between BHL cases and controls, McNemar’s test was used to assess differences in the presence of congenital anomalies (≥1 anomaly vs. none) identified during the neonatal period within each site. For unmatched comparisons and subgroup analyses of the BHL groups (types of congenital anomalies), Pearson’s χ^2^ test or Fisher’s exact test (where expected cell counts were <5) were used as appropriate.

To account for the multi-centre design, stratified analyses were performed by study site. The Mantel–Haenszel method was used to estimate pooled odds ratios (ORs) and confidence intervals (CIs) for the association between BHL and congenital anomalies, adjusting for site. Homogeneity of odds ratios between sites was assessed using the Breslow–Day test.

Changes in the number and distribution of congenital anomalies between birth and 2-years were analysed using χ^2^ tests. Where multiple anatomical systems were compared, Bonferroni correction was applied to account for multiple testing.

Multivariable logistic regression was used to explore factors independently associated with moderate-to-severe NDI at 2-years. The dependent variable was NDI (moderate-severe vs. normal-mild), defined using developmental assessment scores. Independent variables were selected *a priori* based on clinical relevance and included BHL (primary exposure), presence of ≥1 congenital anomaly at 2-years, and study site. Adjusted ORs with 95% CIs were reported. Model fit was assessed using the likelihood ratio test, and explanatory power was evaluated using Nagelkerke R^2^.

A two-sided *p*-value of <0.05 was considered statistically significant.

## 3 Results

### 3.1 Cohort characteristics—sample size and matching.

Demographic data for BHL cases (*n* = 94) and matched controls (*n* = 94) from both sites are shown in Table 1. Birthweight, gestational age, and sex were recorded for BHL cases (*n* = 44) and matched controls (*n* = 44) at site 1 and BHL cases (*n* = 50) and controls (*n* = 50) at site 2. No significant differences in gestational age or birth weight were observed between BHL cases and matched controls (matched 1:1) at either site (Mann-Whitney U test, *p* > 0.05). Sex distribution was also similar between groups at both sites (Chi-squared test, *p* > 0.05).

**TABLE 1:** Demographics of cohorts.

Site	Sample size n	Gestational age (weeks) median (range)	Birthweight (g) median (range)	Sex *N* (%) females
Site 1 BHL	*n* = 44	32 (24–41)	1370 (540–3640)	19 (43%)
Matched controls	*n* = 44	32 (23–41)	1385 (521–3650)	20 (45%)
Site 2 BHL	*n* = 50	32 (23–42)	1680 (475–4360)	19 (38%)
Matched controls	*n* = 50	32 (23–42)	1670 (500–4366)	18 (36%)

At Site 1: Eight children had mild BHL (20–40 dB HL), 24 had moderate BHL (41–70 dB HL), four had severe BHL (71–95 dB HL), and eight had profound BHL (>95 dB HL). At Site 2: Five children had mild BHL, 19 had moderate BHL, 16 had severe BHL and one child had profound BHL. Data on the degree of BHL were missing for *n* = 9 cases at Site 2, all audiological data were complete at Site 1.

### 3.2 Congenital anomalies identified during the neonatal period

**FIGURE 1:**
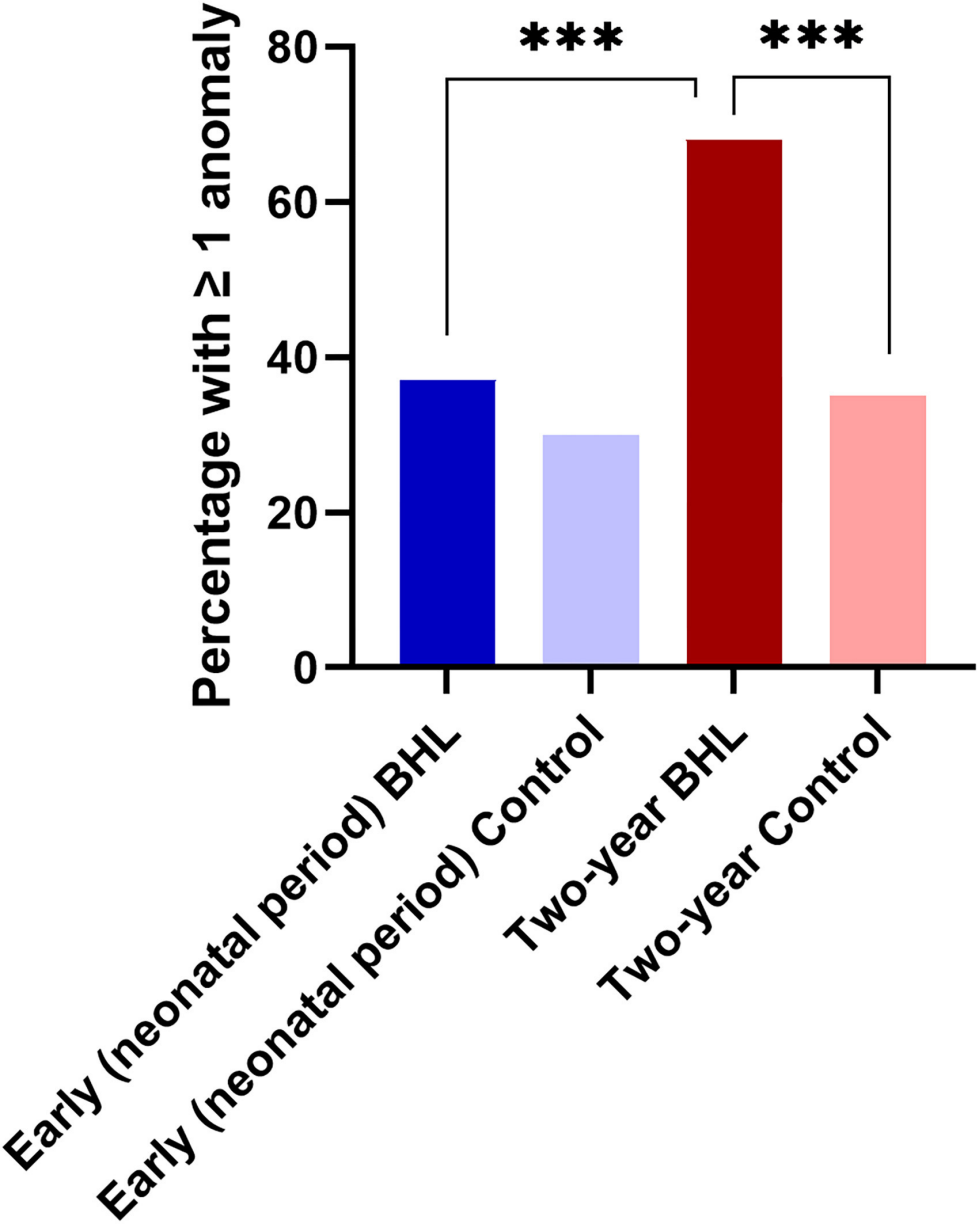
Prevalence of early (neonatal period) congenital anomalies and at 2-years in children with bilateral hearing loss (BHL) and matched NICU controls (no hearing loss). *Y*-axis indicates percentage of children with one or more congenital anomaly for children with bilateral hearing loss (BHL - dark bars) and matched controls (pale bars), matched on gestational age, birthweight and sex. There was no statistical difference between BHL and controls during the early, neonatal period, however at 2-year follow-up, congenital anomalies (≥1) were more frequent among children with BHL compared with matched controls at both sites. When data from both sites were combined, 64/94 (68%) children with BHL had at least one congenital anomaly compared with 33/94 (35%) controls (χ^2^ = 20.47, *p* < 0.001). Stratified analysis (Mantel–Haenszel) demonstrated a significant association between BHL and the presence of congenital anomalies at 2-years (pooled OR 4.43, 95% CI 2.39–8.20, *p* < 0.001, *n* = 188). ***p < 0.001.

The distribution of congenital anomaly counts did not differ significantly between BHL cases and matched controls at either site (Fisher’s Exact test, *p* > 0.05; Supplementary Table 1). When the outcome was simplified to the presence of at least one anomaly, there was no significant difference between matched pairs at either site (McNemar test, *p* > 0.05; Table 2). When data from both centres were combined, there remained no significant association between BHL and early (neonatal period) congenital anomalies (χ^2^ = 1.54, *p* = 0.215, *n* = 188, 94 matched pairs, Figure 1). There was no evidence of heterogeneity between sites (Breslow–Day χ^2^ = 1.17, *p* = 0.493), indicating consistency across cohorts. Stratified by site analyses using the Mantel–Haenszel method confirmed the absence of an association (χ^2^ = 1.17, *p* = 0.28, *n* = 188, 94 matched pairs), with a pooled OR of 1.47 (95% CI 0.80–2.71).

**TABLE 2:** Presence of ≥1 congenital anomaly identified during the neonatal period in bilateral hearing loss (BHL) cases and matched controls.

Site	BHL ≥1 anomaly	BHL none	Matched control ≥1 anomaly	Matched control none	Statistical test	*p*-value
Site 1	18/44	26/44	12/44	32/44	McNemar	*p* = 0.238
Site 2	17/50	33/50	16/50	34/50	McNemar	*p* = 0.824

During the early neonatal period (site 1), one anomaly of the ear was detected; this was included within head and neck anomalies (*n* = 7), as there was also a cleft lip and palate. At 2 years (site 1), additional ear anomalies were identified (*n* = 4), including bilateral auditory canal atresia, a pit at the root of the left helix, a narrowed ear canal, and enlarged vestibular aqueducts. There was only one genetic diagnosis in these cases, DFNB1 deafness due to a pathogenic variant in GJB2.

During the neonatal period (site 2), a greater number of ear abnormalities were detected (*n* = 6), including left folded ear, small ears, abnormally shaped left ear, low-set posteriorly rotated ears, malformation of the left ear, and left ear microtia. Most cases (5/6) had additional head and neck anomalies. At 2 years, no further cases of ear anomalies were reported (*n* = 5). In site 2, genetic or syndromic findings were documented in four of these six cases. Genetic diagnoses of pathological conditions, including linear skin defects with multiple congenital anomalies 3 (LSDMCA3) due to a pathogenic variant in *NDUFB11;* CHARGE syndrome due to a *de novo* likely pathogenic variant in *CHD7*, a *de novo* variant in *TRRAP*, and VATER association on the basis of genetic exclusion.

### 3.3 Anomalies at 2-years

At 2-year follow-up, congenital anomalies (≥1) were more frequent among children with BHL compared with matched controls at both sites (Figure 1). At Site 1, anomalies were present in 34/44 (77%) children with BHL and 17/44 (39%) controls (χ^2^ = 15.01, *p* < 0.001). At Site 2, anomalies were identified in 30/50 (60%) children with BHL and 16/50 (32%) controls (χ^2^ = 9.09, *p* = 0.003). Supplementary Table 2 presents the number of congenital anomalies identified at two years of age, stratified by study site, for children with BHL and matched controls without hearing loss.

When data from both sites were combined, 64/94 (68%) children with BHL had at least one congenital anomaly compared with 33/94 (35%) controls (χ^2^ = 20.47, *p* < 0.001, effect size, Cramer’s *V* = 0.33).

Stratified analysis using the Mantel–Haenszel method demonstrated a significant association between BHL and the presence of congenital anomalies at 2-years (pooled OR 4.43, 95% CI 2.39–8.20, *p* < 0.001, *n* = 188). There was no evidence of heterogeneity between sites (Breslow–Day *p* = 0.41).

### 3.4 Anatomical systems affected

Differences in the distribution of anatomical anomaly categories between birth and 2-years were explored descriptively (Supplementary Table 3).

Exploratory analyses of anatomical anomaly subtypes demonstrated an overall increase in abnormalities between birth and 2-year follow-up, particularly in the nervous system and head and neck. After adjustment for multiple comparisons, only abnormalities of the nervous system remained statistically significant (χ^2^ = 23.2, *p* < 0.001, effect size, Cramer’s *V* = 0.35). Other observed differences were not statistically significant after correction for multiple comparisons.

### 3.5 Predicting neurodevelopmental impairment

Multivariable logistic regression analysis was performed to examine exploratory factors associated with moderate-to-severe NDI at 2-years of age (see Table 3). After adjustment for congenital anomalies at 2-years and study site, BHL was significantly associated with increased odds of moderate-to-severe NDI (adjusted OR 13.89, 95% CI 6.27–30.74, *p* < 0.001, *n* = 188).

**TABLE 3:** Multivariable logistic regression analysis of factors associated with moderate-to-severe neurodevelopmental impairment at 2-years.

Variable	Adjusted OR	95% CI	*p*-value
Bilateral hearing loss	13.89	(6.27–30.74)	<0.001
≥1 Congenital anomalies at 2 years	2.57	(1.14–5.78)	0.023
Study site	5.15	(2.25–11.74)	<0.001

The presence of ≥1 congenital anomaly at 2-years was also independently associated with moderate-to-severe NDI (adjusted OR 2.57, 95% CI 1.14–5.78, *p* = 0.023). Study site was associated with variation in NDI outcomes (adjusted OR 5.15, 95% CI 2.25–11.74, *p* < 0.001).

The overall model was statistically significant (likelihood ratio χ^2^ = 75.24, *p* < 0.001) and explained a moderate proportion of variance in NDI (Nagelkerke R^2^ = 0.45).

### 3.6 Genetics—exploratory descriptive findings

Genetic testing was not performed systematically and was typically undertaken where there was clinical suspicion of a syndromic or inherited cause. Genetic data were available for a subset of children with BHL (Site 1: *n* = 32; Site 2: *n* = 20) and are presented descriptively only. Because genetic testing was not performed uniformly across participants or sites, no formal statistical analysis was undertaken. Clinically reported genetic or syndromic findings were documented in four children at Site 1 and 11 children at Site 2, including pathogenic or likely pathogenic variants, copy number variants, clinical syndromic diagnoses, variants of uncertain significance, and carrier states. For a summary see Supplementary Table 4 of the Supplementary Data Sheet.

## 4 Discussion

This study suggests that congenital anomalies are not differentially recorded between infants with BHL and matched NICU controls at birth, but that substantial group differences in identified anomalies are evident by early childhood follow-up. By 2-years of age, children with BHL exhibit a markedly greater burden of congenital anomalies, particularly involving the nervous system, alongside higher rates of NDI. These findings are consistent with hearing loss in NICU populations reflecting a broader developmental vulnerability rather than an isolated sensory impairment. The temporal divergence observed between birth and follow-up suggests that clinically relevant differences may not be fully recognised, documented, or phenotypically apparent in the neonatal period and reinforces the importance of longitudinal surveillance in this high-risk group.

### 4.1 Mechanisms

Several mechanisms may explain why congenital anomalies are more frequently identified during follow-up in children with BHL. Delayed recognition is likely to contribute, particularly for neurological abnormalities that may not be clinically apparent in the neonatal period. Infants with hearing loss can undergo more intensive surveillance, including imaging and specialist follow-up, increasing the likelihood of identifying previously unrecognised conditions. Although increased surveillance may contribute to this difference, it is unlikely to fully account for the magnitude and pattern of differences observed. Current UK guidance does not routinely include hearing loss alone as an indication for enhanced neonatal follow-up (NICE, 2018).

An alternative explanation is that hearing loss and congenital anomalies may reflect shared developmental processes arising from early disruption to multiple organ systems during critical periods of foetal development. In this framework, hearing loss may serve as a potential early clinical marker of broader neurodevelopmental vulnerability. This aligns with growing evidence that neonatal morbidity may reflect complex, multisystem processes involving genetic factors, inflammatory pathways, and perinatal exposures, which may become increasingly clinically apparent or diagnostically recognised over time (Perrone and Buonocore, 2017; Prasad et al., 2021; Reiss et al., 2022).

One possible explanation for these findings is that hearing loss and congenital anomalies reflect shared developmental or genetic processes affecting multiple organ systems. However, alternative explanations, including differential clinical surveillance and unmeasured confounding, must also be considered. Importantly, these findings should not be interpreted as evidence that congenital anomalies develop postnatally. Rather, they likely reflect a combination of delayed clinical recognition, evolving phenotypic expression, documentation practices, and possible differential ascertainment due to greater clinical surveillance in children with BHL.

### 4.2 Implications for clinical practice

These findings have important implications for clinical practice and the design of neonatal follow-up services. Current approaches to neonatal developmental follow-up often rely on risk factors identified in the neonatal period; however, our results suggest that this may underestimate developmental vulnerability that may not be apparent from neonatal risk factors alone. The increased burden of congenital anomalies and NDI in children with hearing loss highlights the potential role of hearing outcomes as an early clinical marker to inform more targeted, proportionate follow-up. Infants with hearing loss may benefit from enhanced multidisciplinary surveillance to support earlier identification of co-occurring conditions and optimise intervention pathways. Embedding such risk-stratified approaches within health services could improve early identification, support more efficient allocation of resources, and facilitate more targeted intervention. These findings support a move toward longitudinal, data-driven models of care that better reflect the multisystem nature of neonatal risk.

An additional consideration in this population is Auditory Neuropathy Spectrum Disorder (ANSD), characterised by impaired neural synchrony despite preserved outer hair cell function, and represents a potential mechanism linking early auditory dysfunction with neurodevelopmental outcomes in this population (De Siati et al., 2020).

It is also important to consider forms of auditory dysfunction that are not associated with structural anomalies, such as Central Auditory Processing Disorder (CAPD). CAPD reflects impaired neural processing of auditory information despite intact peripheral hearing mechanisms and has been described in preterm and high-risk populations (Amin et al., 2015). However, CAPD is typically diagnosed later in childhood using specialised behavioural assessments and would not be expected to be identified in infancy or at 2-year follow-up. As such, this study does not capture this dimension of auditory pathway dysfunction, which may represent an additional manifestation of neurodevelopmental vulnerability.

### 4.3 Strengths and weaknesses

This study has several strengths. The multi-centre design with matched controls allows for consistent comparisons across independent NICU populations and enhances the generalisability of findings. The inclusion of longitudinal follow-up to 2-years provides important insight into developmental trajectories that would not be captured in neonatal-only studies. Clinically verified outcomes, including formal audiological diagnoses and documented developmental assessments, further strengthen the robustness of the data.

However, several limitations should be acknowledged. The retrospective design relies on routinely collected clinical data and may be subject to incomplete recording and residual confounding. A key limitation is the potential for ascertainment bias. Children with BHL are more likely to undergo enhanced clinical surveillance, including specialist review and additional investigations, which may increase the likelihood of identifying congenital anomalies that might otherwise remain undetected in matched controls. Increased surveillance may also include neuroimaging and genetic or syndromic evaluation, which increases the likelihood of identifying associated anomalies. This differential intensity of follow-up may therefore contribute, at least in part, to the higher prevalence of anomalies observed at 2-years. Although this possibility cannot be excluded, current UK follow-up pathways do not routinely use hearing loss alone as a criterion for enhanced surveillance, and the consistency of findings across both sites suggests that ascertainment bias is unlikely to fully explain the observed differences.

Although this is a multi-centre study, the sample size remains modest. This limits statistical power, particularly for subgroup analyses, and reduces the extent to which findings can be generalised. The regression findings should therefore be interpreted as associations after partial adjustment within a clinically complex NICU population, rather than evidence of an independent effect of hearing loss. The multivariable regression model should therefore be interpreted as exploratory, and external validation in larger prospective cohorts is required.

An additional limitation of the multivariable analysis is the restricted set of covariates included in the model. NICU populations are exposed to a wide range of risk factors for NDI, including prematurity-related brain injury (e.g., intraventricular haemorrhage and periventricular leukomalacia), hyperbilirubinaemia, hypoxic-ischaemic insult, congenital infections such as CMV, and genetic or syndromic conditions. These factors were not uniformly available within the dataset and could not be included in the model. As a result, residual confounding is likely, and the observed association between BHL and NDI should be interpreted as an association after partial adjustment rather than evidence of an independent causal relationship. It is therefore possible that hearing loss reflects underlying neurological or systemic vulnerability rather than acting as an independent driver of NDI. Although both BHL and congenital anomalies were included in the regression model, these factors are likely to be interrelated and may reflect shared underlying developmental or genetic pathways. As such, it is not possible to determine the primary driver of NDI within this cohort, and the observed associations should be interpreted as reflecting overlapping contributions rather than independent causal effects.

A key limitation of this study is the incomplete and non-uniform assessment of aetiologies, particularly with respect to genetic and syndromic causes of hearing loss. Genetic testing was undertaken in a subset of children based on clinical indication, meaning that the prevalence of underlying genetic conditions is likely underestimated and cannot be compared between groups. This is particularly important given that syndromic and genetic conditions are well-established causes of BHL and are frequently associated with multisystem abnormalities and NDI. The co-occurrence of hearing loss, congenital anomalies, and NDI observed in this study may therefore, in part, reflect underlying genetic or syndromic aetiologies that were not fully captured. Future prospective studies incorporating systematic genomic and phenotypic characterisation will be essential to disentangle these relationships and to determine the extent to which shared biological pathways, including genetic factors, contribute to the observed associations.

Furthermore, increased prevalence of congenital anomalies identified at 2-year follow-up is likely influenced, in part, by differences in the timing and method of diagnosis. Some anomalies, particularly those identified through imaging, may not be clinically apparent or routinely investigated in the neonatal period and are therefore more likely to be identified later during follow-up. Given the retrospective nature of the dataset, it was not always possible to distinguish between anomalies present at birth but identified later, and conditions acquired postnatally. As such, some anomalies recorded at 2-year follow-up may reflect delayed diagnosis or postnatal pathology rather than congenital presence.

Congenital anomalies were grouped according to standard classification frameworks to enable analysis; however, individual conditions within these categories are heterogeneous and may differ in their relationship to hearing loss and neurodevelopmental outcomes. This may introduce variability that cannot be fully accounted for within the present analysis.

The increased prevalence of nervous system anomalies identified at 2-year follow-up may reflect a combination of delayed recognition of congenital conditions and the effects of neonatal brain injury or illness. Within a retrospective dataset, it is not always possible to distinguish between congenital anomalies and acquired or evolving neurological abnormalities, and some conditions may be misclassified. This represents an important limitation when interpreting the contribution of neurological findings to later developmental outcomes.

Heterogeneity in developmental assessment methods across sites represents an additional limitation. In particular, the increased use of standardised assessments such as the Bayley Scales at Site 2 may have increased the likelihood of identifying borderline impairment compared with reliance on clinical documentation alone. There is also potential for misclassification of developmental status, particularly where “normal” development was inferred from the absence of documented concerns or referrals rather than formal assessment. While the sample size is appropriate for this population, some subgroup analyses may be underpowered. Differences in local clinical pathways across centres may also influence detection rates; however, the overall consistency of findings between sites supports the robustness of the main results.

A notable study-site effect was observed in the regression model, suggesting potential inter-centre differences that were not fully captured within the current analysis. This may reflect variation in clinical practice, including differences in surveillance intensity, diagnostic pathways, and approaches to developmental assessment (e.g., use of standardised tools versus clinical documentation), as well as potential differences in clinical complexity across sites. As this study was not designed to investigate inter-site variation, these findings should be interpreted cautiously. However, they highlight the importance of standardised multi-centre data collection and harmonised assessment protocols in future studies to improve comparability and reduce variability in outcome ascertainment.

Aetiological classification of hearing loss (e.g., sensorineural, conductive, or mixed) was not uniformly available in this retrospective dataset. As these subtypes differ in underlying mechanism, clinical management, and potential developmental impact, this represents an important limitation and may contribute to heterogeneity in the observed association. While further subgroup characterisation would be valuable, this was not feasible within the constraints of the available data and may risk overinterpretation; this limitation is therefore acknowledged.

Socio-economic factors, including parental education, engagement, and access to resources, are known to influence neurodevelopmental outcomes (Wong et al., 2025). These variables were not available within the present dataset and could not be accounted for in the analysis, representing a potential source of residual confounding.

The findings presented in this study should be interpreted within the context of overlapping developmental, diagnostic, and ascertainment influences that cannot be fully disentangled within this retrospective dataset.

Future research should prioritise prospective, multi-centre longitudinal studies incorporating standardised phenotyping, detailed aetiological and socio-demographic data, and comprehensive audiological assessment. Such approaches will be essential to disentangle congenital and acquired mechanisms, reduce confounding, and refine understanding of developmental risk in this population.

## 5 Conclusion

In conclusion, this multi-centre longitudinal study shows that congenital anomalies are not differentially recorded at birth between NICU infants with and without BHL but are more frequently identified in children with BHL by 2-years of age. Further prospective studies incorporating systematic phenotypic and aetiological assessment will be required to clarify the underlying mechanisms.

Taken together with the observed association between BHL, congenital anomalies, and NDI, these findings are consistent with hearing loss in NICU populations representing a potential clinical marker of broader developmental vulnerability. However, these findings should be interpreted cautiously, as they may also reflect differences in clinical ascertainment and residual confounding.

Longitudinal multidisciplinary follow-up may therefore be important for improving early identification of co-occurring difficulties and informing more targeted care pathways for high-risk infants and their families.

## Data Availability

The original contributions presented in this study are included in the article/Supplementary material, further inquiries can be directed to the corresponding author.
