# Supplementary material for: Congenital anomalies identified during early childhood in NICU infants with bilateral hearing loss: a multi-centre longitudinal cohort study
Source: Front Neurosci. 2026 Jul 22;20:1836937. doi: 10.3389/fnins.2026.1836937 (PMC13437551; doi:10.3389/fnins.2026.1836937)
Supplement: Supplementary file 1 [file Table_1.docx]

**1 Supplementary material**

## **Table 1.** Counting number of congenital anomalies detected at birth by study site for children with bilateral hearing loss (BHL) and matched controls (no hearing loss).

|  | Site 1 | | | Site 2 | |
| --- | --- | --- | --- | --- | --- |
| No. congenital anomalies detected at birth | | **BHL cases** | **Matched controls** | **BHL cases** | **Matched Controls** |
| 0 | | 26/44 (59%) | 32/44 (73%) | 33/50 (66%) | 34/50 (70%) |
| 1 | | 12/44 (27%) | 11/44 (25%) | 8/50 (16%) | 11/50 (20%) |
| 2 | | 3/44(7%) | 1/44 (2%) | 2/50 (4%) | 3/50 (6%) |
| 3 | | 3/44 (7%) | 0/44 (0%) | 2/50 (4%) | 0/50 (0%) |
| 4 or more | | 0/44 (0%) | 0/44 (0%) | 5/50 (10%) | 2/50 (4%) |

**Footnote:** Values are presented as n/N (%).

**Table 2.** Counting number of congenital anomalies detected at 2-years of age by study site for children with bilateral hearing loss (BHL) and matched controls (no hearing loss).

|  | Site 1 | | Site 2 | |
| --- | --- | --- | --- | --- |
| No. physical anomalies detected at 2-years of age | **BHL cases** | **Matched controls** | **BHL cases** | **Matched Controls** |
| 0 | 10/44 (23%) | 27/44 (61%) | 20/50 (40%) | 34/50 (68%) |
| 1 | 12/44 (27%) | 12/44 (27%) | 13/50 (26%) | 6/50 (12%) |
| 2 | 9/44 (20.5%) | 3/44 (7%) | 8/50 (16%) | 5/50 (10%) |
| 3 | 9/44 (20.5%) | 2/44 (5%) | 5/50 (10%) | 3/50 (6%) |
| 4 or more | 4/44 (9%) | 0/44 (0%) | 4/50 (8%) | 2/50 (4%) |

## **Footnote:** Values are presented as n/N (%).

## **Table 3. Comparison of abnormal anatomical features for cases with BHL between birth and two years**

| **Anatomical anomaly** | **No. congenital anomalies at birth**  **Site 1/**  **Site 2** | **Number of congenital anomalies at 2-years**  **Site 1/**  **Site 2** | **Chi-squared value (df), *p* value** | **Effect size**  **P size following Bonferroni correction**  (11 comparisons) |
| --- | --- | --- | --- | --- |
| **Abnormality of head or neck** | 7  10 | 19  12 | χ² (1) = 5.4833  *p* = 0.019 | Cramér's V = 0.1708 (weak)  *p* > 0.05 |
| Abnormality of limbs | 3  2 | 9  2 | *p* > 0.05 |  |
| Growth abnormality | 2  2 | 7  2 | *p* > 0.05 |  |
| Abnormality of the integument | 1  2 | 1  2 | *p* > 0.05 |  |
| **Abnormality of the nervous system** | 6  4 | 16  23 | χ² (1) = 23.2136  *p* = 0.000001 | Cramér's V= 0.3514 (moderate)  *p* = 0.000011 |
| Abnormality of the skeletal system | 2  0 | 3  2 | *p* > 0.05 |  |
| Abnormality of the connective tissue | 0  0 | 0  0 | *p* > 0.05 |  |
| Abnormality of the cardiovascular system | 1  8 | 6  4 | *p* > 0.05 |  |
| Abnormality of the ear | 1  6 | 4  5 | *p* > 0.05 |  |
| Abnormality of the respiratory system | 3  3 | 0  3 | *p* > 0.05 |  |
| Abnormality of the genitourinary system | 3  7 | 9  7 | *p* > 0.05 |  |
| Abnormality of the gastrointestinal system | 2  4 | 3  6 | *p* > 0.05 |  |
| Total number of anomalies | **Site 1: 30**  **Site 2: 42** | **Site 1: 73**  **Site 2: 63** |  |  |

**Footnote:** Statistical comparisons were exploratory and interpreted cautiously due to small sample sizes and multiple comparisons. Data from site 1 and 2 were added to increase sample size. Bonferroni correction was applied across 11 anatomical categories. **Emboldened data** indicates statistically significant difference between the BHL cases and matched controls. Note ear abnormalities are excluded from the total count as these cases are included in abnormality of head or neck since only one abnormality is counted per organ/structure site (see Materials and Methods).

**Table 4. Genetic anomalies**

| **Site** | **Gene / Locus** | **Variant** | **Classification** | **Clinical Interpretation** |
| --- | --- | --- | --- | --- |
| Site 1 | *GJB2 / GJB6* | *GJB2:c.35*del; *GJB6* deletion | Pathogenic (biallelic) | Consistent with genetic hearing loss |
| Site 1 | *GJB2* | *GJB2*:c.101T>C (heterozygous) | Pathogenic (carrier) | Carrier status only |
| Site 1 | *GJB2* | *GJB2*:c.355G>A (heterozygous) | Pathogenic (carrier) | Carrier status only |
| Site 1 | *GJB2* | *GJB2*:c.355G>A (heterozygous) | Pathogenic (carrier) | Carrier status only |
| Site 1 | *GJB2* | *GJB2*:c.35delG; *GJB2*:c.101T>C | Pathogenic (biallelic) | Consistent with genetic hearing loss |
| Site 1 | *FKBP14* | *FKBP14*:c.362dup (homozygous) | Pathogenic | Syndromic connective tissue disorder |
| Site 1 | *CHD7* | *CHD7*:c.4354-6A>G | Variant of Uncertain Significance (VUS) | Uncertain relevance |
| Site 1 | *TECTA* | *TECTA*:c.2239C>T | Variant of Uncertain Significance (VUS) | Uncertain relevance |
| Site 1 | Chromosome 18* | ~17 Mb deletion | Pathogenic CNV | Likely syndromic |
| Site 1 | Chromosome 2* | Gain of material (region not specified) | Variant of Uncertain Significance (VUS) | Uncertain relevance |

| **Site** | **Gene / Locus** | **Variant** | **Classification** | **Clinical Interpretation** |
| --- | --- | --- | --- | --- |
| Site 2 | *NDUFB11** | Not specified | Pathogenic | Mitochondrial disorder |
| Site 2 | Chromosome 9q* | Deletion | Pathogenic CNV | Syndromic |
| Site 2 | *CHD7** | De novo variant (not specified) | Likely pathogenic | CHARGE syndrome |
| Site 2 | *CHD7** | Variant not specified | Pathogenic (clinical diagnosis) | CHARGE syndrome |
| Site 2 | *ZMIZ1* | De novo variant | Pathogenic | Syndromic |
| Site 2 | *BTD** | Not specified | Pathogenic | Biotinidase deficiency |
| Site 2 | *GBA** | Not specified | Pathogenic | Gaucher disease type 2 |
| Site 2 | *TRRAP* | De novo variant | Pathogenic | Syndromic (external ear atresia) |
| Site 2 | *AGL** | Not specified | Pathogenic | Glycogen storage disease type III |
| Site 2 | No pathogenic variant reported* | VATER association | Clinical diagnosis | Syndromic |
| Site 2 | Chromosome 22q11.2 | Microduplication | Pathogenic CNV | Syndromic |

**Footnote:** Gene symbols are reported using HGNC-approved nomenclature and are italicised. Variant descriptions follow HGVS standards and are not italicised. Clinical classifications are based on available reports and ACMG/AMP criteria where provided.

In some cases*, predominately at Site 2, variant-level detail was not recorded in clinical notes and was therefore unavailable due to the retrospective nature of data extraction. Carrier states (heterozygous variants in recessive genes, e.g. *GJB2*) do not confirm a diagnosis of genetic hearing loss. Variant classification reflects clinical documentation at the time of data extraction and was not reinterpreted for this study. Genetic testing was not performed uniformly across all participants; findings are therefore presented descriptively.

At the time of data collection at Site 1 in the cohort with BHL there was genetic testing in thirty-two children. Four identified pathogenic variants causative of deafness, three were VUS, and three identified carrier statuses of recessive conditions. In Site 2 there was genetic testing in twenty children with BHL, and eleven tests identified pathogenic variants.

**5. 0 Specific Diagnoses Included in Each Sub-Group of Abnormal Anatomical Features**

Diagnoses in *italics* were identified by a consultant neonatologist as being congenital.

Inner ear malformations:

- *Cochlear nerve aplasia*
- *Absent auditory nerve*
- *Enlarged vestibular aqueduct*
- *Narrow ear canal*
- *Eustachian tube dysfunction*
- *Vestibulocochlear nerve palsy*
- *Meatal atresia*

Craniofacial abnormalities:

Minor

- *Pre-auricular skin tag*
- *Cyst on ear*
- *Cheek skin tag*
- *Cryptotia*
- *Plagiocephaly*
- *Dysmorphic facial features*
- *Creases in ear lobes*
- *Small close set eyes*
- *Small mouth*
- *Facial asymmetry*
- *Abnormal head shape*
- *Cyst behind ear*
- *Short and upslanting palpebral fissures*
- *Posteriorly rotated ears*
- *Retrognathia*
- *Small chin*

Major

- *Malformation of ear*
- *Unilateral cleft lip*
- *Microtia*
- *Facial nerve palsy*
- *Cyst back of skull*

Neurological and spinal:

- *Craniosynostosis*
- Periventricular flare
- Seizure
- Encephalitis
- Hydrocephalus
- *Wide sagittal suture*
- *Large anterior fontanelle*
- *Choroid plexus cysts*
- *Sacral hemivertebrae*
- *Abnormal cervical vertebral bodies*
- *Butterfly vertebrae*
- *Cerebral Palsy*

Gastrointestinal:

- *Oesophageal atresia*
- *Tracheoesophageal fistula*
- *Oesophageal stricture*
- *Inguinal hernia*
- Umbilical hernia
- Necrotising enterocolitis (GI)

Vision and eye:

- Nystagmus
- Vision impairment
- Long sighted
- Astigmatism
- *Esotropia*
- *Microtropia*
- Chalazion
- Retinopathy of prematurity

Other malformations:

- *Sacral dimple*
- *Hypospadias*
- *Equinovalgus malformation*
- *Single umbilical artery*
- *Dysplastic hip*

Cardiac:

- *Ventricular septal defect*
- *Atrial septal defect*
- *Patent foramen ovale*
- Patent ductus arteriosus (unless diagnosed by a cardiac specialist as this is very common in NICU infants)
- *Tetralogy of fallot*

Respiratory:

- *Congenital lobar emphysema*
- Chronic lung disease
- Bronchopulmonary dysplasia
- *Small upper airway/ Laryngomalacia*
- *Tracheal stenosis*
- Meconium aspiration syndrome
- Congenital pneumonia

Metabolic:

- Neonatal hypoglycaemia
- Hyperinsulinism
- *Hypothyroidism*
- *Pituitary cyst*
- Congenital lactic acidosis

Renal:

- *Small kidney*
- *Vesico-uteric reflux*
- Neonatal acute renal failure
- *Renal cyst*
- *Lobulated and enlarged kidney*

Neuromotor:

- Increased tone in limbs
- *Hypermobility*
- Frequent trips and falls
- Motor co-ordination difficulties
- Mobility issues
- Head lag
- Hypotonia
